# An alternative approach to contrast-enhanced imaging: diffusion-weighted imaging and T_1_-weighted imaging identifies and quantifies necrosis in Wilms tumour

**DOI:** 10.1007/s00330-018-5907-z

**Published:** 2018-12-17

**Authors:** Harriet J. Rogers, Martijn V. Verhagen, Susan C. Shelmerdine, Christopher A. Clark, Patrick W. Hales

**Affiliations:** 1 Developmental Imaging and Biophysics Section, Great Ormond Street Institute of Child Health, University College London, 30 Guilford Street, London, WC1N 1EH UK; 2 Department of Radiology, Great Ormond Street Hospital For Children NHS Foundation Trust, London, WC1N 3JH UK

**Keywords:** Magnetic resonance imaging, Diffusion, Neoplasm, Necrosis, Gadolinium

## Abstract

**Objectives:**

Volume of necrosis in Wilms tumour is informative of chemotherapy response. Contrast-enhanced T_1_-weighted MRI (T_1_w) provides a measure of necrosis using gadolinium. This study aimed to develop a non-invasive method of identifying non-enhancing (necrotic) tissue in Wilms tumour.

**Methods:**

In this single centre, retrospective study, post-chemotherapy MRI data from 34 Wilms tumour patients were reviewed (March 2012–March 2017). Cases with multiple b value diffusion-weighted imaging (DWI) and T_1_w imaging pre- and post-gadolinium were included. Fractional T_1_ enhancement maps were generated from the gadolinium T_1_w data. Multiple linear regression determined whether fitted parameters from a mono-exponential model (ADC) and bi-exponential model (IVIM – intravoxel incoherent motion) (*D*, D*, *f)* could predict fractional T_1_ enhancement in Wilms tumours, using normalised pre-gadolinium T_1_w (T_1_w_norm_) signal as an additional predictor. Measured and predicted fractional enhancement values were compared using the Bland-Altman plot. An optimum threshold for separating necrotic and viable tissue using fractional T_1_ enhancement was established using ROC.

**Results:**

*ADC* and *D* (diffusion coefficient) provided the strongest predictors of fractional T_1_ enhancement in tumour tissue (*p* < 0.001). Using the ADC-T_1_w_norm_ model (adjusted *R*^*2*^ = 0.4), little bias (mean difference = − 0.093, 95% confidence interval = [− 0.52, 0.34]) was shown between predicted and measured values of fractional enhancement and analysed via the Bland-Altman plot. The optimal threshold for differentiating viable and necrotic tissue was 33% fractional T_1_ enhancement (based on measured values, AUC = 0.93; sensitivity = 85%; specificity = 90%).

**Conclusions:**

Combining ADC and T_1_w imaging predicts enhancement in Wilms tumours and reliably identifies and measures necrotic tissue without gadolinium.

**Key Points:**

*• Alternative method to identify necrotic tissue in Wilms tumour without using contrast agents but rather using diffusion and T*_*1*_ *weighted MRI.*

*• A method is presented to visualise and quantify necrotic tissue in Wilms tumour without contrast.*

*• The proposed method has the potential to reduce costs and burden to Wilms tumour patients who undergo longitudinal follow-up imaging as contrast agents are not used.*

## Introduction

Wilms tumour is the most common type of paediatric renal tumour, accounting for approximately 90% of all kidney tumours [1]. In Europe, patients are treated under the SIOP approach (Société Internationale d′Oncologie Pédiatrique), in which they undergo pre-operative chemotherapy to reduce tumour size prior to surgery [2].

Necrosis within Wilms tumour post-chemotherapy is informative of treatment response, particularly when tumour size remains stable. It has been suggested that patients with 100% necrosis post-chemotherapy, when assessed via histological analysis, are associated with relapse-free survival at 5-year follow-up [3]. Thus, quantifying the degree of necrosis in Wilms tumour tissue is beneficial. In the body, MRI can identify necrotic tissue via administration of gadolinium-based contrast agents and T_1_-weighted imaging (T_1_w), where absent or decreased enhancement may represent necrosis. However, gadolinium requires venous access and raises examination costs. In addition, recent reports have described gadolinium retention in neural and body tissue regardless of renal function; however, currently, there are no known sequelae related to this [4]. While, gadolinium is still frequently administered and has many additional uses, an alternative approach to identify and quantify necrosis would be beneficial.

The apparent diffusion coefficient (ADC), derived from diffusion-weighted imaging (DWI), has been shown to be related to cell density; low ADC values correlate with high cell counts in a range of paediatric body tumours [5]. ADC values have been shown to increase following chemotherapy in abdominal tumours [6] and specifically in Wilms tumour [7]. Thus, areas of necrosis in Wilms tumour could potentially be identified as regions with low cellular density, which can result in higher ADC values. However, lower ADC values do not necessarily indicate viable tissue; necrosis by coagulation results in low ADC values which mimics high cellular density tissue [8]. However, hyper-intense regions on pre-gadolinium T_1_w images can indicate areas of coagulated blood; thus, we hypothesise combining ADC and pre-gadolinium T_1_w may enable necrosis in Wilms tumour to be identified and quantified, without the need for exogenous contrast agents.

Furthermore, research has suggested that alternative non-Gaussian diffusion models for DWI, such as intravoxel incoherent motion (IVIM) [9], provide a more accurate description of the diffusion MR signal and provide additional information about tissue-microstructure compared to the standard mono-exponential model [10]. IVIM could be particularly beneficial in assessing necrosis, as it accounts for the influence of blood flow on the DWI signal [11], which should be absent in necrotic tissue.

In this study, we hypothesise that the combination of T_1_w imaging and DWI could estimate the degree of necrosis in Wilms tumour, as opposed to the more traditional method of using gadolinium. We also investigate whether diffusion parameters from IVIM could improve this estimation. Additionally, we aim to establish an upper threshold of enhancement, based on typical values found in necrotic tumour tissue, as defined by manual delineation of necrotic tumour regions on post-gadolinium T_1_ images by two radiologists. Tissue which shows enhancement below this threshold (using either measured or predicted enhancement data) can be classified as necrotic, allowing the quantification of the volume fraction of necrotic tissue in future Wilms tumour studies.

## Materials and methods

### Study population

Institutional ethical approval was granted and waived the need for consent for this single centre study. A 5-year retrospective review (March 2012–2017) of the radiology imaging system (RIS) was performed for all MRI abdominal studies in children with proven histological diagnosis of Wilms tumour at our institution. Inclusion criteria were children who had completed a full 6-week course of chemotherapy, with MRI sequences that included both DWI and T_1_w sequences (pre- and post-gadolinium contrast). Cases where the post-chemotherapy size of the tumour did not cover more than two axial slices on diffusion imaging were excluded. Eight patients from our cohort have previously been reported in [7] although this did not focus on necrosis detection.

### MRI

All imaging was performed on a 1.5 T Siemens Magnetom Avanto scanner equipped with 40 mT/m gradients. Depending on patient size, one or two body matrix coils were used to obtain full coverage (6 element design, Siemens). All patients underwent DWI followed by T_1_w imaging pre- and post-gadolinium. The DWI protocol was as follows: 7 or 8 *b* values in three orthogonal directions (0, 50, 100, 250, 500, 750, 1000 s/mm^2^ (8 Wilms tumours) or 0, 50, 100, 150, 200, 250, 500, 1000 s/mm^2^ (29 Wilms tumour)); slice thickness, 6 mm; TR/TE, 2600 ms/89 ms; and field of view, 350 mm. Axial T_1_-weighted imaging was acquired both before and after intravenous administration of gadolinium-based contrast using identical protocols for the pre- and post-gadolinium acquisitions. The full imaging parameters of all clinical sequences used can be found in [6].

### Contrast agents

All patients received Dotarem 0.5 mmol/ml (manufactured by Guerbet), dosage 0.2 ml per kg body-weight. The post-contrast T_1_ sequence was started 2 to 4 min after injection of contrast agent. Hyoscine butylbromide (Buscopan) 20 mg/ml (manufactured by Sanofi) was also administered prior to all sequences to prevent peristalsis, dosage 0.02 ml per kg body-weight; however, maximum dosage was based on patient age, 1 month–4 years = 0.25 ml maximum and 4 years–12 years = 0.5 ml maximum.

### Post-processing

Data processing and analysis were performed using in-house routines written in Matlab R2015b (MathWorks Inc., Natick). All registrations were performed using NiftyReg [12] packages using affine transformations, and regions of interest (ROIs) were generated using Mango Software (Research Imaging Institute, UTHSCSA).

Tumour ROIs were independently drawn by two radiologists specialising in paediatric radiology (S.S, 4 years paediatric radiology; M.V, 2 years paediatric radiology). ROIs were drawn around the perimeters of each Wilms tumour on *b* = 0 (non-diffusion-weighted) images on each axial slice, using all clinically acquired images for guidance. The overlapping areas (between the two radiologists) were defined as the final Wilms tumour ROIs. ROIs which displayed substantial visual mismatch between the two radiologists were reviewed until consensus was achieved. To compare similarity of size between the independently defined ROIs, the intraclass correlation coefficient (ICC) was calculated.

Data from each patient was processed twice by two different models of diffusion. A mono-exponential fit [Eq. 1] generated ADC; fitting was performed on a voxel-by-voxel basis across all *b* values. A bi-exponential model (IVIM^9^) [Eq. 2] generated the parameters *D* (thermally driven, ‘slow’ diffusion), D* (flow-driven, ‘fast’ diffusion), and *f* (volume fraction associated with ‘fast’ diffusion). In each instance, S(b) is the signal at a given *b* value, and S_0_ is the signal with no diffusion weighting:

1 $$ S(b)={S}_0{e}^{-b. ADC} $$

2 $$ S(b)={S}_0\left(1-f\right){e}^{-b.D}+f{e}^{-b.\left(D+{D}^{\ast}\right)} $$

The fitted parameters (*D*, D*, and *f*) were calculated in a stepwise fashion. Firstly, a linear fit of ln(S/S0) against *b* was calculated at high *b* values (200–1000 s/mm^2^) to determine the value of *D*. Following this, D* and *f* were fit simultaneously using the full *b* value range (with a fixed *D*) to improve the stability of the model fitting process. D* had no constraints on upper boundaries, and *f* was constrained between 0 and 1. This fitting was performed using the Levenberg-Marquardt nonlinear least squares algorithm. All parameter maps were then smoothed with a 2-mm Gaussian kernel.

Fractional enhancement maps were generated from T_1_w scans. Post-gadolinium T_1_w images (post-Gd T_1_w) were registered to pre-gadolinium T_1_w images (pre-Gd T_1_w). All T_1_w scans were smoothed with a 2-mm Gaussian kernel to counteract registration errors. Voxel-wise fractional enhancement maps were calculated, using fractional enhancement = ((post-Gd T_1_w – pre-Gd T_1_w)/(pre-Gd T_1_w)). For example, a fractional enhancement of 0.50 indicates a 50% increase in signal intensity on the T_1_w image following gadolinium administration. Fractional enhancement maps were co-registered to DWI space. Additionally, pre-Gd T_1_w images were normalised to mean pre-Gd T_1_w signal intensity in a reference tissue in each patient, to produce quantitative, normalised pre-Gd T_1_w images (T_1_w_norm_). This was achieved by dividing each pre-Gd T_1_w image by the mean signal intensity in an ROI placed in normal-appearing erector spinae muscles for each patient. These normalised pre-Gd T_1_w images were also registered to the DWI scans.

### Analysis and statistics

Wilms tumour ROIs were placed on co-registered fractional enhancement, diffusion, and T_1_w_norm_ maps. Mean values were calculated for each parameter in every Wilms tumour. The diffusion parameters included ADC and the fitted IVIM parameters (*D*, D*, and *f*). Additionally, the parameter f × D* was investigated. Multiple linear regression was used to calculate the relationship between mean fractional enhancement (dependent variable) and a combination of the mean of a single diffusion parameter and mean T_1_w_norm_ (predictor variables). Statistically significant regression coefficients were defined as having a *p* value < 0.05. Models were then compared based on adjusted *R*^2^ values.

Using the selected regression model, voxel-wise-predicted enhancement maps were generated for each Wilms tumour. Using the Bland-Altman plot, whole tumour values of predicted and measured fractional enhancement were compared, to determine the similarity (confidence intervals) and level of bias (mean difference) between the two techniques.

We also determined an upper threshold for enhancement in necrotic tissue, which would allow tumours to be separated into viable and necrotic components. Regions within each tumour which confidently represented necrosis were independently delineated by the two radiologists, using all clinically acquired MR sequences for guidance. The overlapping areas between the radiologists were defined as the final necrotic ROIs. These were used as the ‘gold-standard’ to represent the necrotic part of each tumour. The remainder of the Wilms tumour was defined as the viable ROI. Both viable and necrotic ROIs were registered onto corresponding measured fractional enhancement maps. The fractional enhancement value of every necrotic and viable voxel was pooled across the cohort. ROC analysis was used to define a fractional enhancement threshold which best separated viable and necrotic tissue based on AUC (area under curve). Tissue within a Wilms tumour with enhancement below this threshold would be classified as necrotic, and tissue with enhancement above this threshold would be classified as having some degree of viability.

## Results

### Study population

**Fig. 1:**
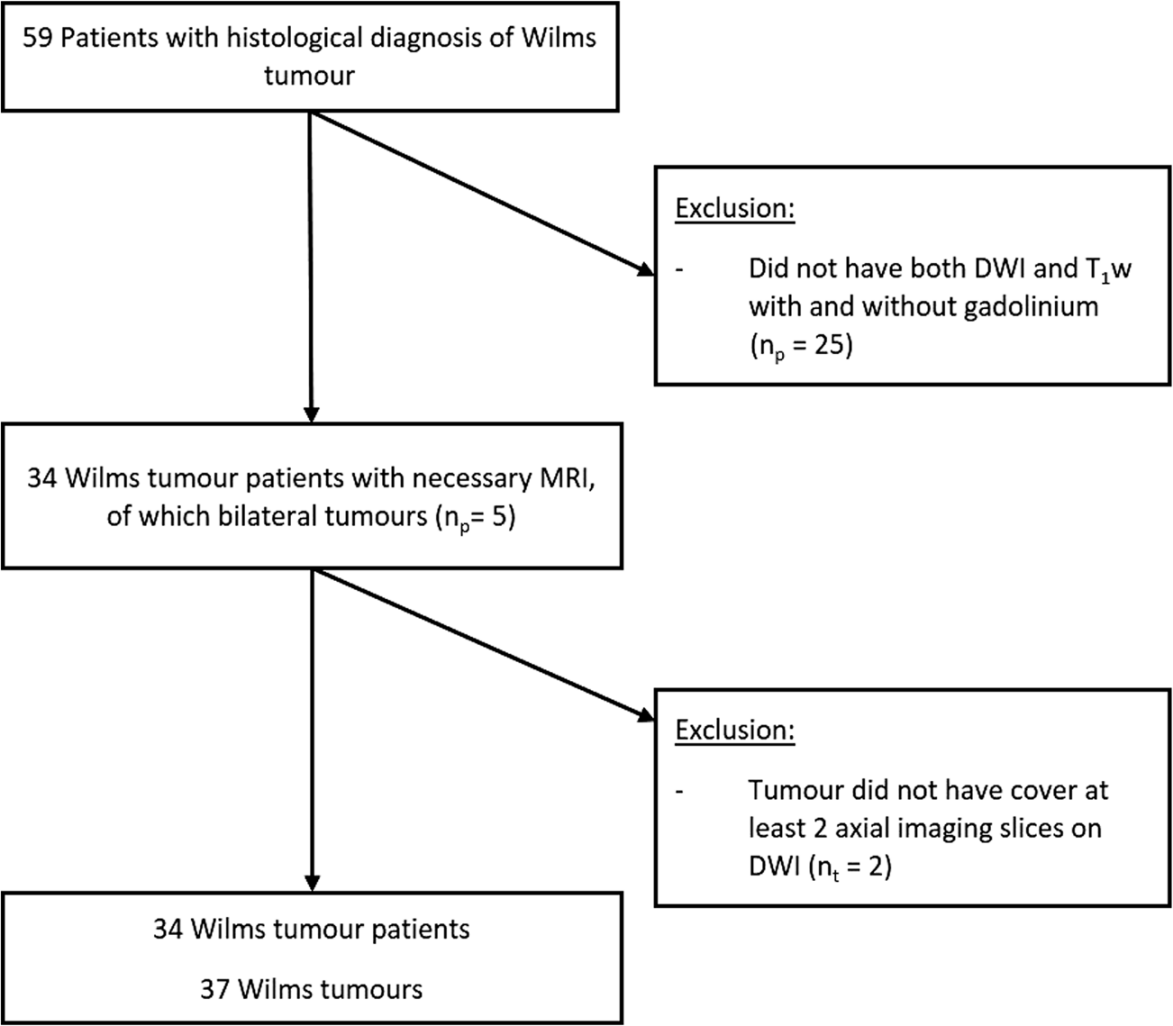
Flowchart of study population showing exclusion criteria. DWI, diffusion-weighted imaging; T_1_w, T_1_ weighted imaging; *n*_*p*_*,* number of patients; *n*_t_, number of tumours

A total of 37 Wilms tumours from 34 patients were included as the final cohort. The median age of patients at the time of their MRI scans was 2.6 years (mean, 3.3 years; SD, 2.6; minimum, 0.4 years; maximum, 11.0 years). Patient inclusion and exclusion metrics are shown in Fig. 1.

### Post-processing

After initial delineation of the Wilms tumour ROIs, visual inspection showed that 8/37 (21.6%) had a substantial mismatch between the radiologists; these were re-defined after consensus. The remaining 29 Wilms tumours had a high level of agreement between radiologists with an average overlapping area of 88% (SD, 0.67). After adjustment of the 8 mismatched ROIs, there was high similarity in the size of the 37 Wilms tumours as defined by the two readers, with an ICC of 0.98 (ICC prior to adjustment, 0.96). There also was high similarity in the size of the necrotic ROIs defined by the two readers, with an ICC of 0.83.

### Analysis

All multiple linear regression models used to predict fractional enhancement were statistically significant (*p* < 0.05), as shown in Table 1.

**Table 1:** The *p* values, *R*^2^, and correlation coefficients (β) of the five multiple regression models used to predict fractional enhancement, based on a combination of T_1_w_norm_ and one of the diffusion parameters. ADC, D, and D* were all measured in standard units of mm/s^2^. f and T_1_w_norm_ are unitless

Diffusion parameter	β_0_ (intercept)	β_1_ (diffusion)	β_2_ (T_1_w_norm_)	Model *p* value	Model adjusted *R*^2^
ADC	1.85	− 408.4	− 0.4	5.7 × 10^−5^	0.40
D (IVIM)	1.83	− 419.64	− 0.4	4.2 × 10^−5^	0.42
D* (IVIM)	1.18	− 1.09	− 0.3	0.017	0.17
f (IVIM)	1.01	1.07	− 0.34	0.025	0.15
f × D*(IVIM)	1.2	− 5.53	− 0.33	0.023	0.15

Mono-exponential fitted parameters: *ADC*, apparent diffusion coefficient. Bi-exponential (IVIM) fitted parameters: *D*, thermally-driven, ‘slow’ diffusion; D*, flow-driven, ‘fast’ diffusion; *f*, volume fraction associated with ‘fast’ diffusion

The combination of *D* from IVIM and T_1_w_norm_ gave the strongest regression model *F*(2, 34) = 13.78, *p* < 0.001, adjusted *R*^*2*^ = 0.42. However, this represented only a very marginal improvement compared to ADC (*F*(2, 34) = 13.2, *p* < 0.001, adjusted *R*^2^ = 0.40). While the other three models all reached significance (*p* < 0.05); the higher *p* values and comparatively low adjusted *R*^2^ values indicated that they did not describe the data as well. Due to the similarity in performance between the regression models based on *D* (IVIM) and ADC (mono-exponential), and the fact that ADC data are more widely acquired clinically, we chose to focus on the ADC-based model for further analysis.

**Fig. 2:**
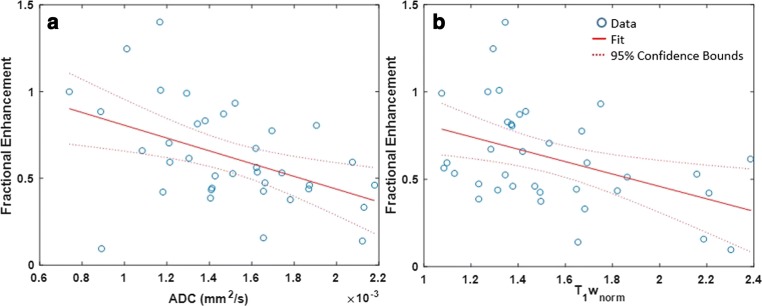
**a** Linear regression of mean ADC (apparent diffusion coefficient) versus mean fractional enhancement in 37 Wilms tumours, adjusted *R*^2^ = 0.19. **b** Linear regression of mean T_1_w_norm_ (normalised quantitative T_1_-weighted imaging) versus mean fractional enhancement in 37 Wilms tumours, adjusted *R*^2^ = 0.16. For the multiple linear regression model (with both ADC and T_1_w_norm_ as predictors), the adjusted *R*^2^ was 0.40 (*p* < 0.001)

Figure 2 demonstrates the relationship between both ADC and T_1_w_norm_ vs. fractional enhancement. Both ADC (*p* < 0.001) and T_1_w_norm_ (*p =* 0.001) added significantly to the prediction, with both increased ADC and increased T_1_w_norm_ being associated with reduced fractional enhancement. The standard error of the estimate was 0.24.

Using the ADC-T_1_w_norm_ model, predicted enhancement was calculated according to the regression model given by Eq. 3, derived the *‘fitlm’ algorithm in Matlab*:

3 $$ \mathrm{Predicted}\ \mathrm{enhancement}=1.85\hbox{--} \left(408.4\times \mathrm{ADC}\right)\hbox{--} \left(0.4\times {\mathrm{T}}_1{\mathrm{w}}_{\mathrm{norm}}\right) $$

where ADC is measured in mm/s^2^.

**Fig. 3:**
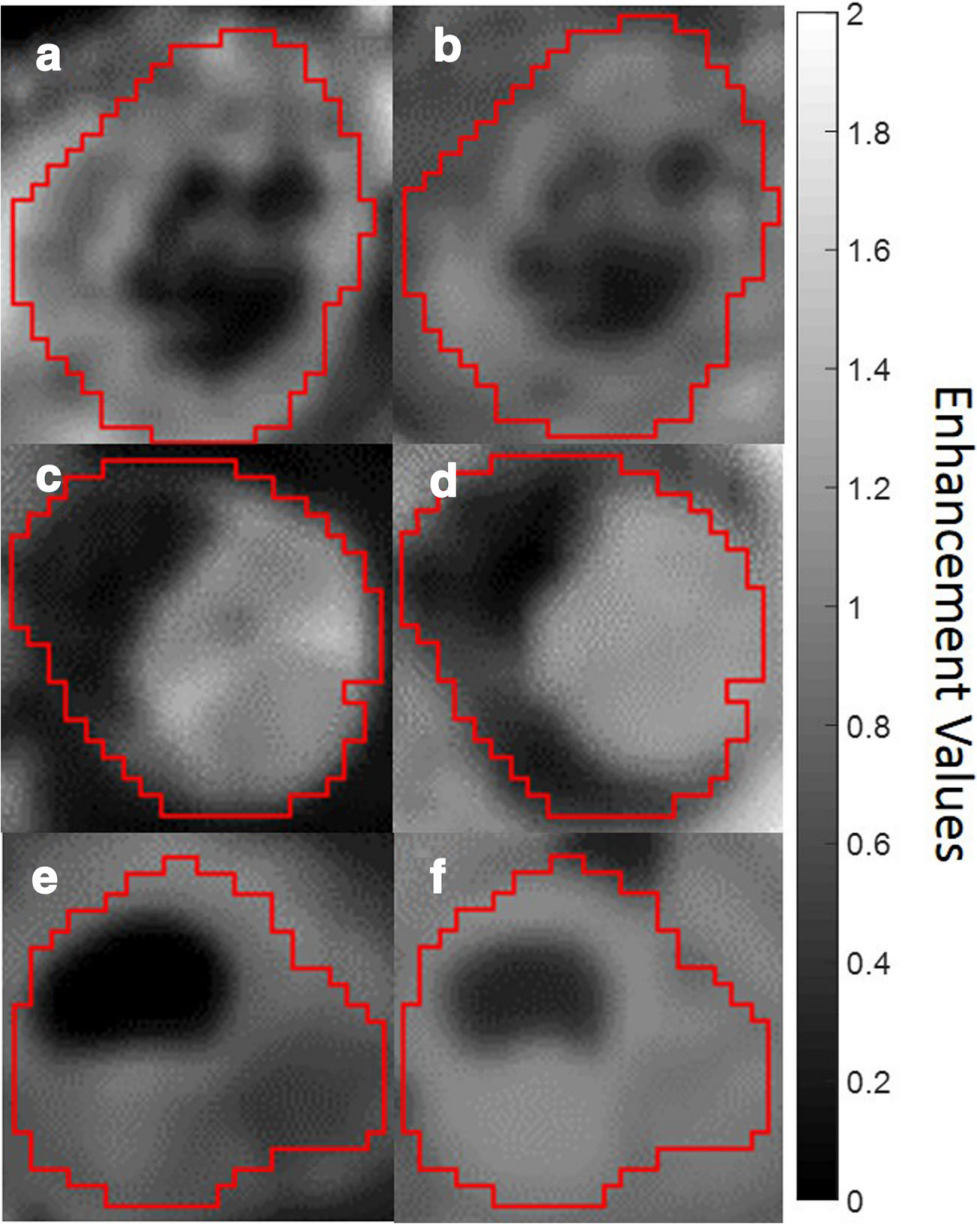
Examples of single axial slices from three representative Wilms tumours. **a**, **c**, **e** Fractional enhancement maps of the Wilms tumours (outlined in red), measured using gadolinium. **b**, **d**, **f** The same slices of the same Wilms tumours from predicted enhancement maps, predicted using Eq. 3 (without gadolinium). Increased signal represents greater enhancement, and hence more viable tissue. Tumour details: A and B—subtype, mixed; age at scan, 11 years. C and D—subtype, blastemal; age at scan, 1.8 years. E and F—subtype, mixed; age at scan, 1.08 years

Comparisons between fractional enhancement and predicted enhancement maps in three representative patients are illustrated in Fig. 3. Both highlight similar regions of enhancing and non-enhancing tissues.

**Fig. 4:**
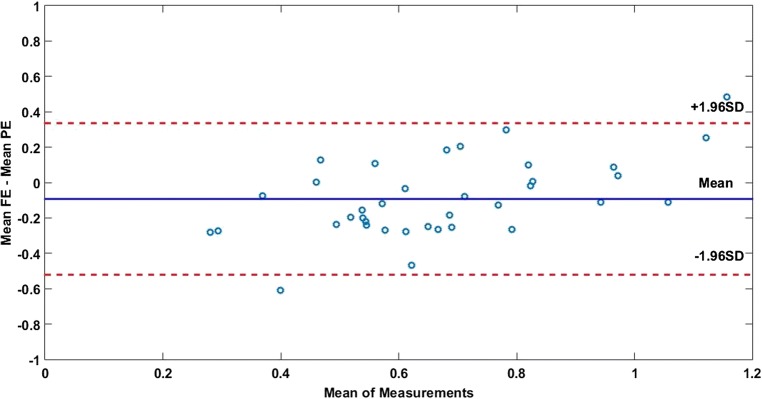
The Bland-Altman plot showing the level of agreement in mean enhancement values in 37 Wilms tumours, as calculated using fractional enhancement (FE) and predicted enhancement (PE) from the ADC-T_1_w_norm_ model (Eq. 3)

The level of agreement between fractional enhancement and predicted enhancement is illustrated in the Bland-Altman plot in Fig. 4*.* There was a slight bias (9%) in predicted values overestimating the level of enhancement across a wide range of enhancement levels (mean difference = − 0.093, 95% CI = [− 0.52, 0.34]).

**Fig. 5:**
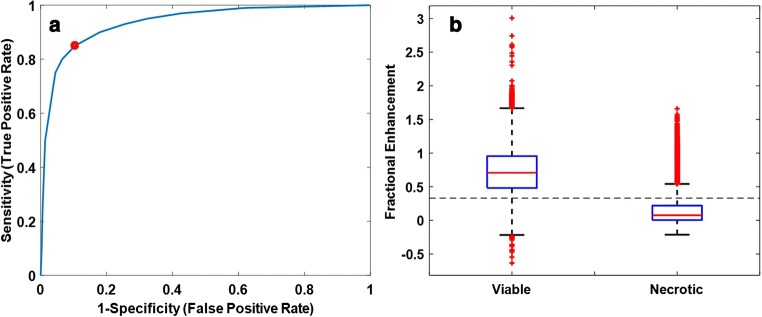
**a** Receiver operator characteristics to determine a threshold which best separates necrotic and viable Wilms tumour tissue. The optimum upper threshold (0.33), whereby voxels displaying enhancement above this value are classified as viable, is highlighted in red. For this threshold, the area under the curve was 0.93, sensitivity was 85%, and the specificity was 90%. **b** Box and whisker plot displaying fractional enhancement of every voxel from the 37 Wilms tumours which were either classified as necrotic or viable. The dotted line reflects the optimum threshold (0.33 fractional enhancement) for this separation based on ROC analysis which is shown in (**a**)

ROC analysis provided an optimal threshold to distinguish between viable and necrotic tissue, based on fractional enhancement (Fig. 5a). The upper threshold was 0.33 (i.e. voxels showing less than 33% signal enhancement on T_1_w imaging, after administration of gadolinium, were classified as necrotic). This threshold provided a sensitivity of 85% and specificity of 90% for identifying the ‘gold-standard’ necrotic tissue, with an AUC of 0.93.

Figure 5b displays a box and whisker plot of the fractional enhancement values in the manually defined necrotic and viable ROIs across the entire cohort. An independent sample *T* test revealed a significant difference between fractional enhancement values in the viable (mean, 0.73; SD, 0.33) and necrotic (mean, 0.14; SD, 0.2) voxels, *t*(195364) = − 446.96, *p* < 0.001. The optimum threshold (0.33) which separates necrotic and viable tumour tissue is also highlighted in Fig. 5b.

## Discussion

This study investigated whether necrosis (non-enhancing tissue) could be identified without using gadolinium contrast-enhanced T_1_w images in Wilms tumour. We found good agreement between mean tumour enhancement values calculated using non-gadolinium-based metrics (ADC and T_1_w_norm_) and the level of enhancement measured using gadolinium in the same tumours. Additionally, a threshold of maximum enhancement in necrotic tissue was determined, which separated viable and necrotic tissue in good agreement with manually delineated necrotic tumour regions, as defined by two specialist paediatric radiologists. As such, this threshold could be used to quantify the total fraction of necrotic tissue in Wilms tumours in future studies, using either measured or predicted enhancement values.

Necrosis within Wilms tumours can indicate chemotherapy response, with high volumes of necrosis representative of ‘good response’ [13]. Quantifying the percentage of necrosis in Wilms tumour has previously been challenging as histological methods usually only sample a sub-section of tissue. Thus, measuring the level of necrosis of the entire tumour volume using imaging-based assessment without exogenous contrast is greatly beneficial. Additionally, in instances of bilateral Wilms tumour, whole tumour resection is not possible and thus necrosis fractions cannot be quantified using histological analysis. Furthermore, DWI and T_1_w imaging are routinely acquired in Wilms tumour patients, so no additional scan time is needed, aiding the transference to clinical practice.

The current gadolinium-based method of identifying non-enhancing tissue has some possible limitations due to potential adverse reactions in patients (including nausea, headaches, and irritation), and the as-yet-unknown impact of the accumulation of contrast agent in patients undergoing repeated follow-up imaging [4]. Additionally, gadolinium may not always be appropriate in Wilms tumour, for example if the patient has renal failure, which will be dependent on co-morbid disorders, tumour staging, treatment timeline, and whether the tumour is bilateral [14, 15]. Additionally, chemotherapy drugs can lead to nephrotoxicity [16]. For these reasons, alternative approaches for predicting enhancement and identifying and quantifying necrotic tissue without gadolinium is potentially beneficial.

ADC is a well-defined diffusion parameter; however, multiple *b* value DWI data allows non-Gaussian diffusion models to be applied, which provide additional fitted parameters. For example, *D* and *f* have shown higher accuracy in distinguishing between enhancing and non-enhancing kidney lesions compared to ADC [17]. Furthermore, bi-exponential models have been suggested rather than mono-exponential for more reliable diffusion estimates of healthy kidney tissue [18]. This study compared ADC and IVIM parameters in predicting fractional enhancement. Interestingly, *f* and *f* × D* regression models did not reach high significance. *f* represents the contribution to the DWI signal due to blood flowing in the randomly orientated capillary network [9], and *f* × D* represents a surrogate measure of blood flow [19]. Due to the lack of blood flow in necrotic tissue, it would be expected that these parameters would be better predictors of fractional enhancement; however, our results suggest this is not the case. This may be because it is beyond the sensitivity of the IVIM model to identify the small level of perfusion in viable Wilms tumours compared to non-enhancing tissue.

*D* produced the strongest regression model; however, the difference between the predictive power of *D* and ADC was minimal. Due to the similarity in the performance of these two predictors and the fact that ADC values are routinely acquired in clinical practice (whereas *D* requires longer, multiple *b* value acquisitions), ADC represents the preferred option for predicting fractional enhancement when combined with T_1_w_norm_. This combination is needed as ADC alone cannot account for necrosis via coagulation, and as can be seen (Fig. 2), the regression is much stronger when T_1_w_norm_ is added as a predictor.

The study had several limitations. Firstly, slightly different *b* values were used for a small number of our patients; however, previous work has shown high reproducibility between ADC values acquired on different scanners with varying *b* values [20]; thus, this is unlikely to influence our analysis. Secondly, when comparing the measured and predicted mean enhancement values, the predicted values were slightly overestimated. However, this bias was small (9%) and may be due to registration errors between T_1_w and ADC maps. Thirdly, our sample size was fairly small, and a more robust model may be possible with a larger cohort. Furthermore, we did not assess tumour necrosis independently using histological methods. However, it is important to note that for histological analysis of Wilms tumour, only a sub-section of the tumour is sampled, and this may not accurately reflect the total necrosis volume. As such, we preferred in this study to use visual assessment of the entire tumour volume, using all clinically available MRI scans, to ensure the entire tumour volume was assessed.

An additional limitation may arise from the possible reliance of our model on the specifics of the T_1_w protocol used in this study. Alterations in the delay time between gadolinium administration and T_1_w imaging may lead to different levels of enhancement on contrast-enhanced T_1_w scans. However, as our model uses the fractional difference between the pre- and post-contrast T_1_w signal, provided the T_1_w protocol remains consistent between these two acquisitions, the influence of variations in the specifics of the T_1_w acquisition between different institutions should mostly cancel out.

Finally, gadolinium is frequently administered for indications broader than necrosis assessment, for example vascular anatomy, and detecting lesions in a variety of organs. We acknowledge that the proposed method cannot entirely replace gadolinium. Despite this, our method would be a suitable alternative for those with severe renal impairment and limit the cumulative gadolinium exposure for patients who have repeated follow-up MRI scans. Gadolinium-free MRI examinations are currently being investigated for paediatric oncology [21], and the proposed model could facilitate this, given that it uses data acquired as part of the clinical standard.

In conclusion, the proposed model predicts enhancement in Wilms tumour without gadolinium and provides a visual representation of tissue viability and necrosis within tumours. A threshold of maximum enhancement in necrotic regions has also been generated, allowing the percentage of necrotic tissue to be quantified in future Wilms tumour studies, using imaging-based methods.

## Abbreviations

D: Diffusion coefficient from IVIM (thermally-driven, ‘slow’ diffusion)
D*: Diffusion parameter from IVIM (flow-driven, ‘fast’ diffusion)
F: Diffusion parameter from IVIM (volume fraction associated with ‘fast’ diffusion)
SIOP: Société Internationale d′Oncologie Pédiatrique (International Society of Paediatric Oncology)
T_1_w_norm_: Normalised pre-gadolinium T_1_-weighted images.

## References

[1] Pastore G, Znaor A, Spreafico F, Graf N, Pritchard-Jones K, Steliarova-Foucher E (2006) Malignant renal tumours incidence and survival in European children (1978–1997): report from the automated childhood cancer information system project. Eur J Cancer 42:2103–2114 10.1016/j.ejca.2006.05.010 16919774

[2] van den Heuvel-Eibrink MM, Hol JA, Pritchard-Jones K (2017). Position paper: rationale for the treatment of Wilms tumour in the UMBRELLA SIOP–RTSG 2016 protocol. Nat Rev Urol.

[3] Boccon-Gibod L, Rey A, Sandstedt B (2000). Complete necrosis induced by preoperative chemotherapy in Wilms tumor as an indicator of low risk: report of the international society of paediatric oncology (SIOP) nephroblastoma trial and study 9. Med Pediatr Oncol.

[4] McDonald RJ, McDonald JS, Kallmes DF (2015). Intracranial gadolinium deposition after contrast-enhanced MR imaging. Radiology.

[5] Humphries PD, Sebire NJ, Siegel MJ, Olsen ØE (2007). Tumors in pediatric patients at diffusion-weighted MR imaging: apparent diffusion coefficient and tumor cellularity. Radiology.

[6] McDonald K, Sebire NJ, Anderson J, Olsen OE (2011). Patterns of shift in ADC distributions in abdominal tumours during chemotherapy-feasibility study. Pediatr Radiol.

[7] Hales PW, Olsen ØE, Sebire NJ, Pritchard-Jones K, Clark CA (2015) A multi-Gaussian model for apparent diffusion coefficient histogram analysis of Wilms’ tumour subtype and response to chemotherapy. NMR Biomed 28:948–957 10.1002/nbm.3337 26058670

[8] LaViolette PS, Mickevicius NJ, Cochran EJ (2014). Precise ex vivo histological validation of heightened cellularity and diffusion-restricted necrosis in regions of dark apparent diffusion coefficient in 7 cases of high-grade glioma. Neuro Oncol.

[9] Le Bihan D, Breton E, Lallemand D, Aubin ML, Vignaud J, Laval-Jeantet M (1988) Separation of diffusion and perfusion in intravoxel incoherent motion MR imaging. Radiology 168:497–505 10.1148/radiology.168.2.3393671 3393671

[10] Yuan J, Yeung DK, Mok GS (2014). Non-Gaussian analysis of diffusion weighted imaging in head and neck at 3T: a pilot study in patients with nasopharyngeal carcinoma. PLoS One.

[11] Iima M, Le Bihan D (2015). Clinical intravoxel incoherent motion and diffusion MR imaging: past, present, and future. Radiology.

[12] Modat M, Cash DM, Daga P, Winston GP, Duncan JS, Ourselin S (2014) Global image registration using a symmetric block-matching approach. J Med Imaging (Bellingham). 10.1117/1.JMI.1.2.024003 10.1117/1.JMI.1.2.024003 PMC4478989 26158035

[13] Godzinski J (2015). The current status of treatment of Wilms’ tumor as per the SIOP trials. J Indian Assoc Pediatr Surg.

[14] Breslow NE, Collins AJ, Ritchey ML, Grigoriev YA, Peterson SM, Green DM (2005) End stage renal disease in patients with Wilms tumor: results from the national Wilms tumor study group and the United States renal data system. J Urol 174:1972–1975 10.1097/01.ju.0000176800.00994.3a PMC1483840 16217371

[15] Grigoriev Y, Lange J, Peterson SM (2012). Treatments and outcomes for end-stage renal disease following Wilms tumor. Pediatr Nephrol.

[16] Perazella MA (2012). Onco-nephrology: renal toxicities of chemotherapeutic agents. Clin J Am Soc Nephrol.

[17] Chandarana H, Lee VS, Hecht E, Taouli B, Sigmund EE (2011) Comparison of biexponential and monoexponential model of diffusion weighted imaging in evaluation of renal lesions: preliminary experience. Invest Radiol 46:285 10.1097/RLI.0b013e3181ffc485 21102345

[18] Zhang JL, Sigmund EE, Chandarana H (2010). Variability of renal apparent diffusion coefficients: limitations of the monoexponential model for diffusion quantification. Radiology.

[19] Le Bihan D, Turner R (1992). The capillary network: a link between IVIM and classical perfusion. Magn Reson Med.

[20] Grech-Sollars M, Hales PW, Miyazaki K (2015). Multi-centre reproducibility of diffusion MRI parameters for clinical sequences in the brain. NMR Biomed.

[21] Muehe AM, Theruvath AJ, Lai L et al (2017) How to provide gadolinium-free PET/MR cancer staging of children and young adults in less than 1 h: the Stanford approach. Mol Imaging Biol. 10.1007/s11307-017-1105-7 10.1007/s11307-017-1105-7 PMC5773418 28721605

